# 3-[(*E*)-3-(2,4-Dichloro­phen­yl)prop-2-en­oyl]-4-hy­droxy-2*H*-chromen-2-one

**DOI:** 10.1107/S1600536810043837

**Published:** 2010-10-31

**Authors:** Mohammad Asad, Chuan-Wei Oo, Hasnah Osman, Ching Kheng Quah, Hoong-Kun Fun

**Affiliations:** a School of Chemical Sciences, Universiti Sains Malaysia, 11800 USM, Penang, Malaysia; b X-ray Crystallography Unit, School of Physics, Universiti Sains Malaysia, 11800 USM, Penang, Malaysia

## Abstract

In the title compound, C_18_H_10_Cl_2_O_4_, the chromen-2-one ring system is almost planar [maximum deviation = 0.028 (1) Å] and is inclined at an angle of 16.35 (4)° with respect to the benzene ring. The C=C bond has an *E* configuration. The mol­ecular conformation is stabilized by an almost symmetric intra­molecular O⋯H⋯O hydrogen bond and a C—H⋯O inter­action, both of which form *S*(6) ring motifs. In the crystal structure, mol­ecules are linked into sheets lying parallel to (100) *via* inter­molecular C—H⋯O hydrogen bonds. The crystal packing is further consolidated by π–π stacking inter­actions [centroid-to-centroid separation = 3.6615 (6) Å].

## Related literature

For general background to and the biological activity of chalcones, see: Claisen *et al.* (1881 ▶); Siddiqui *et al.* (2008 ▶); Harborne & Mabry (1982 ▶); Bandgar *et al.* (2010 ▶). For related structures, see: Arshad *et al.* (2010 ▶); Asad *et al.* (2010 ▶). For bond-length data, see: Allen *et al.* (1987 ▶). For hydrogen-bond motifs, see: Bernstein *et al.* (1995 ▶). For the stability of the temperature controller used for the data collection, see: Cosier & Glazer (1986 ▶).
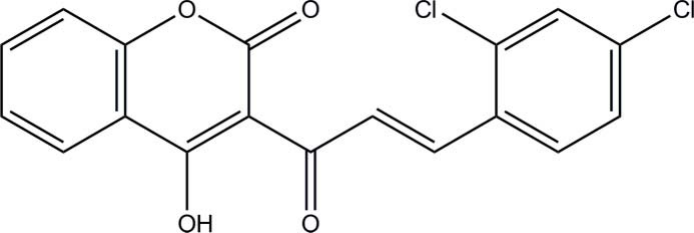


## Experimental

### 

#### Crystal data

C_18_H_10_Cl_2_O_4_
*M* *_r_* = 361.16
Monoclinic, 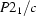

*a* = 4.5233 (2) Å
*b* = 21.2099 (9) Å
*c* = 15.6304 (7) Å
β = 91.607 (1)°
*V* = 1498.97 (11) Å^3^
*Z* = 4
Mo *K*α radiation
μ = 0.45 mm^−1^
*T* = 100 K
0.35 × 0.15 × 0.09 mm

#### Data collection

Bruker SMART APEXII DUO CCD diffractometer
Absorption correction: multi-scan (*SADABS*; Bruker, 2009 ▶) *T*_min_ = 0.857, *T*_max_ = 0.959
25117 measured reflections
6698 independent reflections
5270 reflections with *I* > 2σ(*I*)
*R*_int_ = 0.033

#### Refinement

*R*[*F* ^2^ > 2σ(*F* ^2^)] = 0.036
*wR*(*F* ^2^) = 0.101
*S* = 1.05
6698 reflections
222 parameters
H atoms treated by a mixture of independent and constrained refinement
Δρ_max_ = 0.54 e Å^−3^
Δρ_min_ = −0.34 e Å^−3^

### 

Data collection: *APEX2* (Bruker, 2009 ▶); cell refinement: *SAINT* (Bruker, 2009 ▶); data reduction: *SAINT*; program(s) used to solve structure: *SHELXTL* (Sheldrick, 2008 ▶); program(s) used to refine structure: *SHELXTL*; molecular graphics: *SHELXTL*; software used to prepare material for publication: *SHELXTL* and *PLATON* (Spek, 2009 ▶).

## Supplementary Material

Crystal structure: contains datablocks global, I. DOI: 10.1107/S1600536810043837/hb5700sup1.cif

Structure factors: contains datablocks I. DOI: 10.1107/S1600536810043837/hb5700Isup2.hkl

Additional supplementary materials:  crystallographic information; 3D view; checkCIF report

## Figures and Tables

**Table 1:** Hydrogen-bond geometry (Å, °)

*D*—H⋯*A*	*D*—H	H⋯*A*	*D*⋯*A*	*D*—H⋯*A*
O3—H1O⋯O4	1.27 (2)	1.17 (2)	2.3947 (11)	156 (2)
C11—H11*A*⋯O2	0.93	2.29	2.8704 (12)	120
C4—H4*A*⋯O4^i^	0.93	2.45	3.2514 (13)	144
C17—H17*A*⋯O1^ii^	0.93	2.54	3.3966 (13)	154

Symmetry codes: (i) 
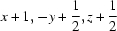
; (ii) 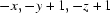.

**Fig. 1.**
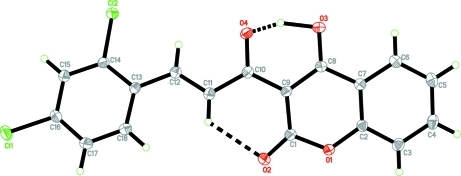
The molecular structure of the title compound showing 50% probability displacement ellipsoids for non-H atoms. Intramolecular interactions are shown in dashed lines.

**Fig. 2.**
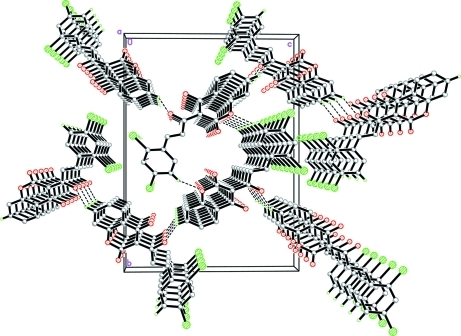
The crystal structure of the title compound, viewed along the a axis. H atoms not involved in hydrogen bonds (dashed lines) have been omitted for clarity.

## supplementary crystallographic information

### Comment

Chalcones are generally prepared from aldehydes and methyl ketones under basic conditions by applying the Claisen–Schmidt condensation (Claisen *et al.*, 1881; Siddiqui *et al.*, 2008). A large number of chalcones and their derivatives are found in natural and synthetic products and are also biogenetically precursors of known flavonoids, isoflavonoids (Harborne & Mabry, 1982) which exhibited a potential variety of biological activities (Bandgar *et al.*, 2010).

In the title molecule, (I), (Fig. 1), the chromen-2-one (O1/C1–C9) ring system is nearly planar (maximum deviation = 0.028 (1) Å for atom C1) and is inclined at an angle of 16.35 (4) ° with the phenyl ring (C13–C18). The C11═C12 bond has an *E* configuration. The molecule is stabilized by intramolecular O3—H1O···O4 and C11—H11A···O2 hydrogen bonds, which form S(6) ring motifs (Bernstein *et al.*, 1995). Bond lengths (Allen *et al.*, 1987) and angles are within normal ranges and comparable with the related structures (Arshad *et al.*, 2010; Asad *et al.*, 2010).

In the crystal packing (Fig. 2), the molecules are linked into two-dimensional sheets parallel to (100) *via* intermolecular C4—H4A···O4 and C17—H17A···O1 hydrogen bonds (Table 1). Short intermolecular distances [3.6615 (6) Å] between symmetry-related O1/C1/C2/C7–C9 (centroid Cg1) and C2–C7 (centroid Cg2) rings [symmetry code: -1+x, y, z] indicate the existence of π–π stacking interactions.

### Experimental

To a stirred solution of 3-acetyl-4-hydroxycoumarin (0.98 mmol, 200 mg) in ethyl alcohol (10 ml), 2,4-dichlorobenzaldehyde (0.98 mmol, 171 mg) was added in the presence of one drop of piperidine. The mixture was refluxed on water bath for 14 h. After cooling at room temperature, a yellow solid was obtained, filtered, washed with ethanol–water, dried and recrystallized from chloroform as shining yellow needles of (I) in 70% yield.

### Refinement

H1O was located in a difference Fourier map and allowed to refined freely. The remaining H atoms were positioned geometrically and refined using a riding model with C—H = 0.93 Å and *U*_iso_(H) = 1.2*U*_eq_(C). The highest residual electron density peak is located at 0.64 Å from C12 and the deepest hole is located at 1.13 Å from Cl2.

### Crystal data

**Table:**

C_18_H_10_Cl_2_O_4_	*F*(000) = 736
*M**_r_* = 361.16	*D*_x_ = 1.600 Mg m^−^^3^
Monoclinic, *P*2_1_/*c*	Mo *K*α radiation, λ = 0.71073 Å
Hall symbol: -P 2ybc	Cell parameters from 9100 reflections
*a* = 4.5233 (2) Å	θ = 2.3–35.1°
*b* = 21.2099 (9) Å	µ = 0.45 mm^−^^1^
*c* = 15.6304 (7) Å	*T* = 100 K
β = 91.607 (1)°	Needle, yellow
*V* = 1498.97 (11) Å^3^	0.35 × 0.15 × 0.09 mm
*Z* = 4	

### Data collection

**Table:**

Bruker SMART APEXII DUO CCD diffractometer	6698 independent reflections
Radiation source: fine-focus sealed tube	5270 reflections with *I* > 2σ(*I*)
graphite	*R*_int_ = 0.033
φ and ω scans	θ_max_ = 35.3°, θ_min_ = 1.6°
Absorption correction: multi-scan (*SADABS*; Bruker, 2009)	*h* = −7→7
*T*_min_ = 0.857, *T*_max_ = 0.959	*k* = −27→34
25117 measured reflections	*l* = −25→22

### Refinement

**Table:**

Refinement on *F*^2^	Primary atom site location: structure-invariant direct methods
Least-squares matrix: full	Secondary atom site location: difference Fourier map
*R*[*F*^2^ > 2σ(*F*^2^)] = 0.036	Hydrogen site location: inferred from neighbouring sites
*wR*(*F*^2^) = 0.101	H atoms treated by a mixture of independent and constrained refinement
*S* = 1.05	*w* = 1/[σ^2^(*F*_o_^2^) + (0.0509*P*)^2^ + 0.2749*P*] where *P* = (*F*_o_^2^ + 2*F*_c_^2^)/3
6698 reflections	(Δ/σ)_max_ = 0.001
222 parameters	Δρ_max_ = 0.54 e Å^−^^3^
0 restraints	Δρ_min_ = −0.34 e Å^−^^3^

### Special details

**Table:**

Experimental. The crystal was placed in the cold stream of an Oxford Cyrosystems Cobra open-flow nitrogen cryostat (Cosier & Glazer, 1986) operating at 100.0 (1) K.
Geometry. All esds (except the esd in the dihedral angle between two l.s. planes) are estimated using the full covariance matrix. The cell esds are taken into account individually in the estimation of esds in distances, angles and torsion angles; correlations between esds in cell parameters are only used when they are defined by crystal symmetry. An approximate (isotropic) treatment of cell esds is used for estimating esds involving l.s. planes.
Refinement. Refinement of F^2^ against ALL reflections. The weighted R-factor wR and goodness of fit S are based on F^2^, conventional R-factors R are based on F, with F set to zero for negative F^2^. The threshold expression of F^2^ > 2sigma(F^2^) is used only for calculating R-factors(gt) etc. and is not relevant to the choice of reflections for refinement. R-factors based on F^2^ are statistically about twice as large as those based on F, and R- factors based on ALL data will be even larger.

### Fractional atomic coordinates and isotropic or equivalent isotropic displacement parameters (Å^2^)

**Table:**

	*x*	*y*	*z*	*U*_iso_*/*U*_eq_	
Cl1	−0.68600 (6)	0.640460 (12)	0.153533 (17)	0.02236 (7)	
Cl2	−0.07973 (6)	0.434153 (13)	0.069793 (16)	0.02180 (7)	
O1	0.69790 (16)	0.35450 (4)	0.57063 (5)	0.01808 (14)	
O2	0.37593 (19)	0.41872 (4)	0.51001 (5)	0.02516 (17)	
O3	0.96509 (16)	0.28446 (4)	0.34161 (5)	0.01797 (14)	
O4	0.63677 (16)	0.35486 (4)	0.26596 (5)	0.01701 (14)	
C1	0.5646 (2)	0.37989 (5)	0.49758 (6)	0.01664 (17)	
C2	0.9014 (2)	0.30666 (5)	0.56843 (6)	0.01535 (17)	
C3	1.0072 (2)	0.28411 (5)	0.64694 (7)	0.01883 (18)	
H3A	0.9402	0.3009	0.6978	0.023*	
C4	1.2149 (2)	0.23600 (5)	0.64775 (7)	0.02067 (19)	
H4A	1.2889	0.2207	0.6998	0.025*	
C5	1.3149 (2)	0.21011 (5)	0.57151 (7)	0.02046 (19)	
H5A	1.4544	0.1779	0.5731	0.025*	
C6	1.2059 (2)	0.23256 (5)	0.49397 (7)	0.01786 (18)	
H6A	1.2708	0.2153	0.4431	0.021*	
C7	0.9967 (2)	0.28162 (5)	0.49195 (6)	0.01467 (16)	
C8	0.87645 (19)	0.30746 (4)	0.41294 (6)	0.01417 (16)	
C9	0.6643 (2)	0.35648 (4)	0.41544 (6)	0.01407 (16)	
C10	0.54393 (19)	0.37953 (5)	0.33533 (6)	0.01447 (16)	
C11	0.3233 (2)	0.42963 (5)	0.32717 (6)	0.01642 (17)	
H11A	0.2750	0.4541	0.3742	0.020*	
C12	0.1915 (2)	0.43961 (5)	0.25011 (6)	0.01545 (17)	
H12A	0.2476	0.4133	0.2058	0.019*	
C13	−0.0299 (2)	0.48736 (5)	0.22909 (6)	0.01444 (16)	
C14	−0.1625 (2)	0.49008 (5)	0.14673 (6)	0.01522 (16)	
C15	−0.3651 (2)	0.53657 (5)	0.12291 (6)	0.01714 (17)	
H15A	−0.4475	0.5379	0.0677	0.021*	
C16	−0.4410 (2)	0.58096 (5)	0.18364 (7)	0.01636 (17)	
C17	−0.3254 (2)	0.57894 (5)	0.26670 (7)	0.01765 (17)	
H17A	−0.3843	0.6082	0.3072	0.021*	
C18	−0.1210 (2)	0.53266 (5)	0.28840 (6)	0.01714 (17)	
H18A	−0.0415	0.5315	0.3439	0.021*	
H1O	0.812 (5)	0.3171 (11)	0.2882 (15)	0.077 (7)*	

### Atomic displacement parameters (Å^2^)

**Table:**

	*U*^11^	*U*^22^	*U*^33^	*U*^12^	*U*^13^	*U*^23^
Cl1	0.02114 (11)	0.01919 (12)	0.02702 (13)	0.00611 (8)	0.00565 (9)	0.00516 (9)
Cl2	0.02795 (12)	0.02120 (13)	0.01605 (11)	0.00727 (9)	−0.00287 (8)	−0.00406 (9)
O1	0.0216 (3)	0.0197 (4)	0.0130 (3)	0.0062 (3)	0.0009 (2)	0.0002 (3)
O2	0.0307 (4)	0.0272 (4)	0.0177 (4)	0.0140 (3)	0.0026 (3)	0.0002 (3)
O3	0.0201 (3)	0.0190 (3)	0.0149 (3)	0.0025 (3)	0.0016 (2)	−0.0027 (3)
O4	0.0179 (3)	0.0202 (4)	0.0130 (3)	0.0002 (2)	−0.0004 (2)	−0.0016 (3)
C1	0.0194 (4)	0.0166 (4)	0.0139 (4)	0.0014 (3)	0.0001 (3)	0.0004 (3)
C2	0.0154 (4)	0.0149 (4)	0.0157 (4)	0.0007 (3)	0.0009 (3)	0.0016 (3)
C3	0.0196 (4)	0.0212 (5)	0.0157 (4)	0.0006 (3)	0.0005 (3)	0.0030 (4)
C4	0.0194 (4)	0.0220 (5)	0.0204 (5)	0.0004 (4)	−0.0016 (3)	0.0068 (4)
C5	0.0181 (4)	0.0179 (5)	0.0254 (5)	0.0025 (3)	0.0000 (3)	0.0038 (4)
C6	0.0169 (4)	0.0161 (4)	0.0206 (4)	0.0007 (3)	0.0016 (3)	−0.0001 (4)
C7	0.0150 (4)	0.0137 (4)	0.0154 (4)	−0.0010 (3)	0.0009 (3)	0.0007 (3)
C8	0.0140 (4)	0.0140 (4)	0.0146 (4)	−0.0021 (3)	0.0009 (3)	−0.0007 (3)
C9	0.0147 (4)	0.0150 (4)	0.0125 (4)	−0.0001 (3)	0.0004 (3)	0.0002 (3)
C10	0.0135 (4)	0.0147 (4)	0.0152 (4)	−0.0027 (3)	−0.0004 (3)	0.0000 (3)
C11	0.0164 (4)	0.0167 (4)	0.0161 (4)	0.0000 (3)	−0.0008 (3)	0.0006 (3)
C12	0.0148 (4)	0.0158 (4)	0.0156 (4)	−0.0012 (3)	−0.0005 (3)	0.0006 (3)
C13	0.0146 (4)	0.0150 (4)	0.0138 (4)	−0.0014 (3)	0.0004 (3)	0.0010 (3)
C14	0.0165 (4)	0.0156 (4)	0.0135 (4)	0.0001 (3)	0.0008 (3)	−0.0009 (3)
C15	0.0173 (4)	0.0181 (5)	0.0160 (4)	0.0013 (3)	−0.0001 (3)	0.0008 (3)
C16	0.0148 (4)	0.0145 (4)	0.0200 (4)	0.0007 (3)	0.0037 (3)	0.0021 (3)
C17	0.0187 (4)	0.0164 (4)	0.0179 (4)	−0.0005 (3)	0.0035 (3)	−0.0017 (3)
C18	0.0179 (4)	0.0181 (4)	0.0154 (4)	−0.0013 (3)	0.0004 (3)	−0.0012 (3)

### Geometric parameters (Å, °)

**Table:**

Cl1—C16	1.7358 (10)	C6—H6A	0.9300
Cl2—C14	1.7376 (10)	C7—C8	1.4436 (13)
O1—C2	1.3711 (12)	C8—C9	1.4162 (13)
O1—C1	1.3851 (12)	C9—C10	1.4371 (13)
O2—C1	1.2059 (12)	C10—C11	1.4610 (13)
O3—C8	1.2910 (11)	C11—C12	1.3457 (13)
O3—H1O	1.27 (2)	C11—H11A	0.9300
O4—C10	1.2851 (12)	C12—C13	1.4554 (13)
O4—H1O	1.17 (2)	C12—H12A	0.9300
C1—C9	1.4595 (13)	C13—C18	1.4050 (14)
C2—C7	1.3877 (14)	C13—C14	1.4061 (13)
C2—C3	1.3895 (14)	C14—C15	1.3896 (13)
C3—C4	1.3869 (15)	C15—C16	1.3870 (14)
C3—H3A	0.9300	C15—H15A	0.9300
C4—C5	1.3988 (16)	C16—C17	1.3867 (14)
C4—H4A	0.9300	C17—C18	1.3841 (14)
C5—C6	1.3804 (15)	C17—H17A	0.9300
C5—H5A	0.9300	C18—H18A	0.9300
C6—C7	1.4062 (13)		
			
C2—O1—C1	122.95 (8)	C10—C9—C1	122.14 (8)
C8—O3—H1O	100.6 (11)	O4—C10—C9	118.16 (9)
C10—O4—H1O	105.2 (12)	O4—C10—C11	117.46 (8)
O2—C1—O1	115.24 (9)	C9—C10—C11	124.38 (9)
O2—C1—C9	127.70 (9)	C12—C11—C10	118.48 (9)
O1—C1—C9	117.06 (8)	C12—C11—H11A	120.8
O1—C2—C7	122.00 (8)	C10—C11—H11A	120.8
O1—C2—C3	116.57 (9)	C11—C12—C13	126.60 (9)
C7—C2—C3	121.43 (9)	C11—C12—H12A	116.7
C4—C3—C2	118.53 (10)	C13—C12—H12A	116.7
C4—C3—H3A	120.7	C18—C13—C14	116.78 (9)
C2—C3—H3A	120.7	C18—C13—C12	122.66 (8)
C3—C4—C5	121.09 (9)	C14—C13—C12	120.56 (9)
C3—C4—H4A	119.5	C15—C14—C13	122.39 (9)
C5—C4—H4A	119.5	C15—C14—Cl2	116.89 (7)
C6—C5—C4	119.76 (9)	C13—C14—Cl2	120.72 (7)
C6—C5—H5A	120.1	C16—C15—C14	118.18 (9)
C4—C5—H5A	120.1	C16—C15—H15A	120.9
C5—C6—C7	119.91 (9)	C14—C15—H15A	120.9
C5—C6—H6A	120.0	C17—C16—C15	121.71 (9)
C7—C6—H6A	120.0	C17—C16—Cl1	119.83 (8)
C2—C7—C6	119.27 (9)	C15—C16—Cl1	118.46 (8)
C2—C7—C8	118.22 (8)	C18—C17—C16	118.89 (9)
C6—C7—C8	122.51 (9)	C18—C17—H17A	120.6
O3—C8—C9	121.89 (9)	C16—C17—H17A	120.6
O3—C8—C7	118.46 (8)	C17—C18—C13	121.97 (9)
C9—C8—C7	119.64 (8)	C17—C18—H18A	119.0
C8—C9—C10	117.78 (8)	C13—C18—H18A	119.0
C8—C9—C1	120.01 (8)		
			
C2—O1—C1—O2	175.23 (9)	O2—C1—C9—C10	1.19 (16)
C2—O1—C1—C9	−4.08 (14)	O1—C1—C9—C10	−179.59 (8)
C1—O1—C2—C7	2.35 (14)	C8—C9—C10—O4	−0.56 (13)
C1—O1—C2—C3	−177.35 (9)	C1—C9—C10—O4	−177.65 (9)
O1—C2—C3—C4	−179.58 (9)	C8—C9—C10—C11	179.82 (9)
C7—C2—C3—C4	0.72 (15)	C1—C9—C10—C11	2.74 (14)
C2—C3—C4—C5	−0.55 (16)	O4—C10—C11—C12	11.55 (13)
C3—C4—C5—C6	−0.03 (16)	C9—C10—C11—C12	−168.83 (9)
C4—C5—C6—C7	0.47 (15)	C10—C11—C12—C13	−179.31 (9)
O1—C2—C7—C6	−179.98 (9)	C11—C12—C13—C18	4.13 (15)
C3—C2—C7—C6	−0.30 (15)	C11—C12—C13—C14	−176.17 (9)
O1—C2—C7—C8	0.23 (14)	C18—C13—C14—C15	2.61 (14)
C3—C2—C7—C8	179.92 (9)	C12—C13—C14—C15	−177.10 (9)
C5—C6—C7—C2	−0.31 (14)	C18—C13—C14—Cl2	−176.94 (7)
C5—C6—C7—C8	179.47 (9)	C12—C13—C14—Cl2	3.35 (13)
C2—C7—C8—O3	179.79 (9)	C13—C14—C15—C16	−1.12 (14)
C6—C7—C8—O3	0.01 (14)	Cl2—C14—C15—C16	178.44 (7)
C2—C7—C8—C9	−0.80 (13)	C14—C15—C16—C17	−1.44 (14)
C6—C7—C8—C9	179.43 (9)	C14—C15—C16—Cl1	178.24 (7)
O3—C8—C9—C10	1.20 (13)	C15—C16—C17—C18	2.35 (15)
C7—C8—C9—C10	−178.19 (8)	Cl1—C16—C17—C18	−177.32 (8)
O3—C8—C9—C1	178.35 (9)	C16—C17—C18—C13	−0.73 (15)
C7—C8—C9—C1	−1.04 (13)	C14—C13—C18—C17	−1.66 (14)
O2—C1—C9—C8	−175.83 (10)	C12—C13—C18—C17	178.05 (9)
O1—C1—C9—C8	3.39 (13)		

### Hydrogen-bond geometry (Å, °)

**Table:**

*D*—H···*A*	*D*—H	H···*A*	*D*···*A*	*D*—H···*A*
O3—H1O···O4	1.27 (2)	1.17 (2)	2.3947 (11)	156 (2)
C11—H11A···O2	0.93	2.29	2.8704 (12)	120
C4—H4A···O4^i^	0.93	2.45	3.2514 (13)	144
C17—H17A···O1^ii^	0.93	2.54	3.3966 (13)	154

Symmetry codes: (i) *x*+1, −*y*+1/2, *z*+1/2; (ii) −*x*, −*y*+1, −*z*+1.
